# ESCRT Machinery Mediates Cytokinetic Abscission in the Unicellular Red Alga *Cyanidioschyzon merolae*

**DOI:** 10.3389/fcell.2020.00169

**Published:** 2020-04-03

**Authors:** Fumi Yagisawa, Takayuki Fujiwara, Tokiaki Takemura, Yuki Kobayashi, Nobuko Sumiya, Shin-ya Miyagishima, Soichi Nakamura, Yuuta Imoto, Osami Misumi, Kan Tanaka, Haruko Kuroiwa, Tsuneyoshi Kuroiwa

**Affiliations:** ^1^Center for Research Advancement and Collaboration, University of the Ryukyus, Okinawa, Japan; ^2^Graduate School of Engineering and Science, University of the Ryukyus, Okinawa, Japan; ^3^Department of Gene Function and Phenomics, National Institute of Genetics, Shizuoka, Japan; ^4^JST-Mirai Program, Japan Science and Technology Agency, Saitama, Japan; ^5^Department of Genetics, The Graduate University for Advanced Studies, Shizuoka, Japan; ^6^Laboratory for Chemistry and Life Science, Institute of Innovative Research, Tokyo Institute of Technology, Yokohama, Japan; ^7^School of Life Sciences and Technology, Tokyo Institute of Technology, Yokohama, Japan; ^8^Laboratory of Cell and Functional Biology, Faculty of Science, University of the Ryukyus, Okinawa, Japan; ^9^Department of Cell Biology, Johns Hopkins University School of Medicine, Baltimore, MD, United States; ^10^Department of Biological Science and Chemistry, Faculty of Science, Yamaguchi University, Yamaguchi, Japan; ^11^Graduate School of Sciences and Technology for Innovation, Yamaguchi University, Yamaguchi, Japan; ^12^Department of Chemical and Biological Science, Japan Women’s University, Tokyo, Japan

**Keywords:** ESCRT, cytokinesis, cytokinetic abscission, red alga, *Cyanidioschyzon merolae*

## Abstract

In many eukaryotes, cytokinesis proceeds in two successive steps: first, ingression of the cleavage furrow and second, abscission of the intercellular bridge. In animal cells, the actomyosin contractile ring is involved in the first step, while the endosomal sorting complex required for transport (ESCRT), which participates in various membrane fusion/fission events, mediates the second step. Intriguingly, in archaea, ESCRT is involved in cytokinesis, raising the hypothesis that the function of ESCRT in eukaryotic cytokinesis descended from the archaeal ancestor. In eukaryotes other than in animals, the roles of ESCRT in cytokinesis are poorly understood. To explore the primordial core mechanisms for eukaryotic cytokinesis, we investigated ESCRT functions in the unicellular red alga *Cyanidioschyzon merolae* that diverged early in eukaryotic evolution. *C. merolae* provides an excellent experimental system. The cell has a simple organelle composition. The genome (16.5 Mb, 5335 genes) has been completely sequenced, transformation methods are established, and the cell cycle is synchronized by a light and dark cycle. Similar to animal and fungal cells, *C. merolae* cells divide by furrowing at the division site followed by abscission of the intercellular bridge. However, they lack an actomyosin contractile ring. The proteins that comprise ESCRT-I–IV, the four subcomplexes of ESCRT, are partially conserved in *C. merolae*. Immunofluorescence of native or tagged proteins localized the homologs of the five ESCRT-III components [charged multivesicular body protein (CHMP) 1, 2, and 4–6], apoptosis-linked gene-2-interacting protein X (ALIX), the ESCRT-III adapter, and the main ESCRT-IV player vacuolar protein sorting (VPS) 4, to the intercellular bridge. In addition, ALIX was enriched around the cleavage furrow early in cytokinesis. When the ESCRT function was perturbed by expressing dominant-negative VPS4, cells with an elongated intercellular bridge accumulated—a phenotype resulting from abscission failure. Our results show that ESCRT mediates cytokinetic abscission in *C. merolae*. The fact that ESCRT plays a role in cytokinesis in archaea, animals, and early diverged alga *C. merolae* supports the hypothesis that the function of ESCRT in cytokinesis descended from archaea to a common ancestor of eukaryotes.

## Introduction

**FIGURE 1:**
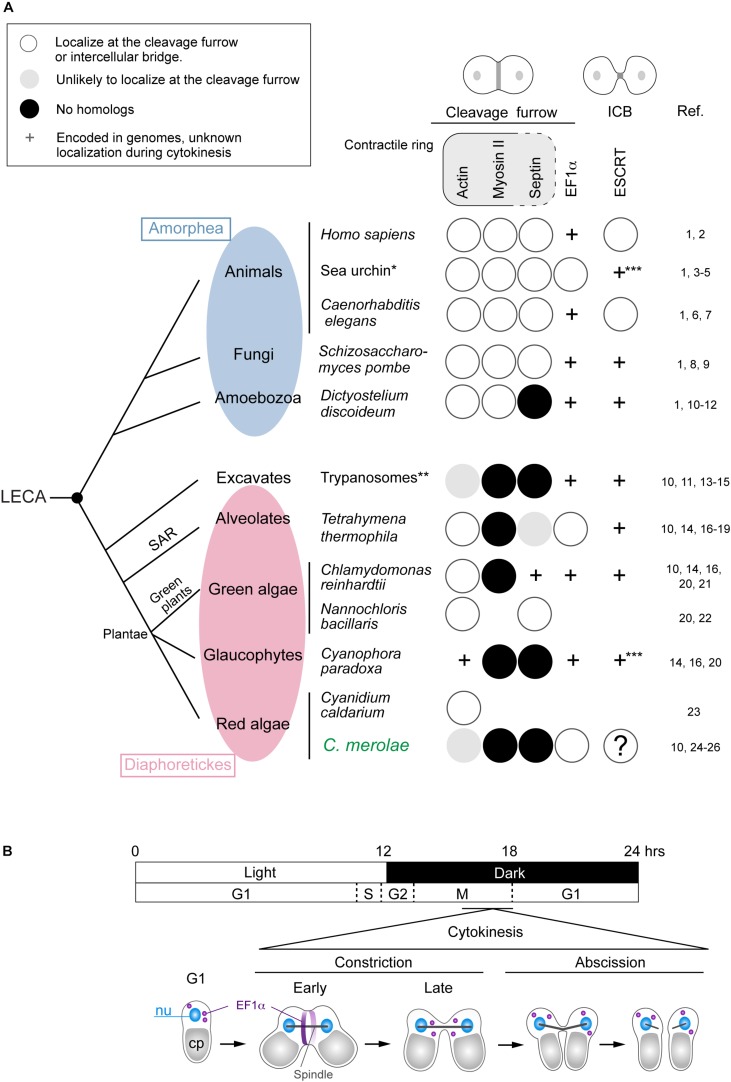
Distribution of representative proteins in the contractile ring, EF1α, and ESCRT in eukaryotes that divide centripetally and a scheme for *C. merolae* cytokinesis. **(A)** The phylogenetic tree is based on Burki (2014). Branch length does not represent evolutionary distance. Amorphea generally divide depending on the actomyosin contractile ring. Other eukaryotes lack myosin-II, an essential component of the contractile ring. Septins are part of the contractile ring in animal cells, whereas in fungal cells, they form separate ring structures. Some contractile ring components are found in the intercellular bridge (ICB), which is not depicted in the figure. Protein localization (to the cleavage furrow or intercellular bridge) or the presence of genes in species were investigated by literature and/or BLAST searching.BLAST searching was conducted using the following protein sequences as the query: *Saccharomyces cerevisiae* Act1 for *C. paradoxa* actin, *Saccharomyces cerevisiae* septins (Cdc3, Cdc10, Cdc11, Cdc12, and Shs1) for *C. merolae* septins, *S. cerevisiae* Tef1 for EF1α in the species with “ + ” marks in the EF1α column, and *S. cerevisiae* Vps2 and Vps4 for ESCRT in sea urchin and *Cyanophora*. References are listed on the right: 1, Pollard, 2017; 2, Campsteijn et al., 2016; 3, Fujimoto and Mabuchi, 2010; 4, Henson et al., 2017; 5, Otto and Schroeder, 1990; 6, Carvalho et al., 2009; 7, Green et al., 2013; 8, Iwaki et al., 2007; 9, Wu et al., 2010; 10, Leung et al., 2008; 11, Nishihama et al., 2011; 12, Reichl et al., 2008; 13, Garcia-Salcedo et al., 2004; 14, Sebe-Pedros et al., 2014; 15, Zhou et al., 2014; 16, Pasha et al., 2016; 17, Hosein et al., 2003; 18, Numata et al., 2000; 19, Wloga et al., 2008; 20, Yamazaki et al., 2013; 21, Cross and Umen, 2015; 22, Yamamoto et al., 2007; 23, Suzuki et al., 1995; 24, Imoto et al., 2011; 25, Matsuzaki et al., 2004; 26, Takahashi et al., 1995. ^∗^Information from several sea urchin species, including *Strongylcentrotus purpuratus* and *Hemicentrotus pulcherrimus*, was combined. ^∗∗^Information from *Trypanosoma brucei* and *Trypanosoma cruzi*. ^∗∗∗^Both Vps2 and Vps4 homologs were found. Blank, the genome information is unavailable or incomplete. LECA, the last eukaryotic common ancestor. **(B)** A 12 h/12 h light and dark cycle synchronized *C. merolae* cell division. Cytokinesis occurs in the dark period after the nuclear division. EF1α accumulates at the cleavage furrow in the early constriction stage and becomes dispersed in the late constriction stage. Cleavage of the intercellular stage occurs at the abscission stage.

Cytokinesis is a fundamental biological phenomenon in all organisms. However, in eukaryotes the mechanisms are diverse. A significant difference exists between a group of animals, fungi, and Amoebozoa (Amorphea; Burki, 2014) and the other groups (Excavates and Diaphoretickes) (Figure 1A). Cells of Amorphea generally divide depending on constriction of the contractile ring (Pollard, 2017; Figure 1A), whereas those of other eukaryotic groups lack myosin-II, an essential ring component (Mishra et al., 2013; Figure 1A). The mechanism of cytokinesis has varied further in each group during evolution. One example is the cytokinesis of land plants whose cells divide by developing cell walls and membranes from the cell center toward the cell periphery (Muller and Jurgens, 2016). Our current knowledge of cytokinesis mostly depends on a limited number of model organisms. However, the mechanisms in different lineages warrant exploration to reveal the eukaryotic history and the core mechanisms of cytokinesis shared by eukaryotes.

In mammalian cells, cytokinesis proceeds by equatorial membrane furrowing followed by abscission of the intercellular bridge. The contractile ring containing actomyosin and septin filaments constricts to furrow the membrane (Green et al., 2012). As the ring closes, the midbody, the platform for the abscission machinery where the plus and minus ends of spindle microtubules overlap, is formed. The actin-capping protein that controls actin polymerization is required for the process (Terry et al., 2018). The intercellular bridge contains the spindle midzone microtubules and midbody. The septin filaments are reorganized into rings in the early intercellular bridge to assist bridge maturation (Renshaw et al., 2014; Karasmanis et al., 2019).

The endosomal sorting complex required for transport (ESCRT), a protein complex conserved among eukaryotes (Table 1), contributes to various membrane fusion/fission events such as multivesicular body formation at late endosomes and nuclear envelope fusion (Campsteijn et al., 2016; Schoneberg et al., 2017). In mammalian cells, ESCRT mediates scission of the intercellular bridge (Carlton and Martin-Serrano, 2007; Morita et al., 2007; Elia et al., 2011; Guizetti et al., 2011; Campsteijn et al., 2016; Schoneberg et al., 2017). The proteins comprising ESCRT-I–IV, the four subcomplexes of ESCRT, are sequentially targeted to the midbody. ESCRT-I recruits charged multivesicular body protein (CHMP) 4 in ESCRT-III by binding to CHMP6 in ESCRT-III by itself or through ESCRT-II (Christ et al., 2016). CHMP6 is a nucleation factor for ESCRT-III. A recent study showed that Septin (SEPT) 9, a constituent of the septin ring, associates with the ESCRT-I protein tumor susceptibility gene (TSG) 101 to assist the recruitment of ESCRT-II and demarcate the sites for ESCRT-III assembly (Karasmanis et al., 2019). The septin ring disassembles as ESCRT-III machinery develops (Karasmanis et al., 2019). In addition to ESCRT-I, the ESCRT-III adaptor protein apoptosis-linked gene-2-interacting protein X (ALIX) localizes at the midbody to separately recruit CHMP4 without binding to CHMP6 (Christ et al., 2016).

**TABLE 1:** Major ESCRT and ESCRT-associated proteins in eukaryotes and archaea.

		Eukaryotes			Archaea	
		
	Mammals	*Saccharomyces*	*C. merolae*	*Sulfolobus*	*Sulfolobus*	Asgard
		*cerevisiae*		*acidocaldarius*	*islandicus*	archaea
ESCRT-I	TSG101	Vps23	**TSG101/CMK136C**			+ (Steadiness box)
	VPS28*	Vps28	VPS28/CMN120C			+
	VPS37A–D	Vps37	-			
	MVB12A, B	Mvb12	-			
ESCRT-II	EAP20	Vps25	EAP20/CMM195C			+
	EAP30*	Vps22	EAP30/CMO296C			+ (Vps22/36-like)
	EAP45	Vps36	-			
ESCRT-III	CHMP1A, B	Did2/Vps46	**CHMP1/CMQ376C**	CHMP-like:	CHMP-like:	+ (Vps2/24/46-like)
			**CHMP1/CMR340C**	CdvB,	ESCRT-III,	
	CHMP2A, B	Vps2	**CHMP2/CMB008C**	Saci_0451,	ESCRT-III-1,	
	CHMP3	Vps24	-	Saci_1416,	ESCRT-III-2,	
	CHMP4A-C	Vps32/Snf7	**CHMP4/CMI044C**	Saci_1601	ESCRT-III-3	+ (Vps20/32/60-like)
	CHMP5	Vps60	**CHMP5/VIG1/CML153C**			
	CHMP6	Vps20	**CHMP6/CMQ184C**			
	CHMP7	-	-			
	IST1	Ist1	-			
ESCRT-IV	VPS4A, B	Vps4	**VPS4/CMO281C**	Vps4/CdvC	Vps4/CdvC	+
	LIP5	Vta1	LIP5/CMI268C			
ALIX	ALIX	Bro1	**ALIX/CMC051C**			+ (Bro1 domain)
CdvA	-	-	-	CdvA	CdvA	

*The list of proteins in mammals and *S. cerevisiae* excluding CdvA is based on Schuh and Audhya (2014). In the *C. merolae* genome, ESCRT and ALIX homologs were searched by BLAST using *S. cerevisiae* sequences as queries. The results for ESCRT homologs were consistent with those of Leung et al. (2008). CdvA homologs in eukaryotes were searched by BLAST using the *S. acidocaldarius* sequence as a query. “−” no detectable homologs. The information of *Sulfolobus* is based on Lindas et al. (2008); Samson et al. (2008), and Liu et al. (2017). Both *Sulfolobus* species have four CHMP family proteins. It is unclear which eukaryotic CHMP protein is the closest. “+” indicates that the homologous sequence is present in the genomes of “Asgard” archaea, the group proposed to be the closest to eukaryotes (Zaremba-Niedzwiedzka et al., 2017). Blank indicates that the protein or gene is not mentioned in the above results. Underlined proteins localize at the midbody or intercellular bridge (mammals) or between daughter nucleoids (*Sulfolobus*). The references are in this legend or the text. ^∗^VPS28 and EAP30 are required for midbody localization of TSG101 and EAP20, respectively (Christ et al., 2016). Proteins in bold font were examined in this study.*

ESCRT-III consists of CHMP family proteins, which are homologous to each other, and increased sodium tolerance (IST) 1 (Table 1). They are coiled-coil proteins suggested to polymerize into spiral filaments beneath the intercellular bridge membrane to narrow abscission sites adjacent to the midbody (Guizetti et al., 2011; Mierzwa et al., 2017; Goliand et al., 2018). ESCRT-III also recruits the microtubule-severing enzyme spastin (Yang et al., 2008; Connell et al., 2009). The intercellular bridge is cleaved after the arrival of vacuolar protein sorting (VPS) 4, the AAA-ATPase in ESCRT-IV, which regulates the turnover of ESCRT-III assembly (Carlton and Martin-Serrano, 2007; Morita et al., 2007; Elia et al., 2011; Schuh and Audhya, 2014; Mierzwa et al., 2017).

ESCRT possibly represents conserved machinery in eukaryotic cytokinesis inherited from the archaeal ancestor. In *Sulfolobus*, a thermophile archaeon, homologs of ESCRT-III proteins and VPS4, and the ESCRT-III scaffold cell division protein (Cdv) A are detected between daughter nucleoids of dividing cells, correlating with the site of membrane ingression (Table 1; Lindas et al., 2008; Samson et al., 2008, 2011; Liu et al., 2017). They are necessary for cytokinesis from early to final stages. Whereas ESCRT-dependent cytokinesis is not universal in archaea (Makarova et al., 2010), recent studies support that eukaryotes have diverged from archaea encoding ESCRT (Zaremba-Niedzwiedzka et al., 2017; Table 1). However, in eukaryotes other than in animals, whether ESCRT mediates cytokinetic abscission is poorly understood. In the land plant *Arabidopsis thaliana*, *elc* mutation, a mutation of TSG101, results in the production of multinucleated cells (Spitzer et al., 2006). Although the mechanism underlying induction of the phenotype is unclear, it may reflect conserved functions of ESCRT in eukaryotic cytokinetic abscission. Some similarities between the animal midbody and plant phragmoplasts, arrays of microtubules on the division plane, have been indicated in a previous study (Otegui et al., 2005).

Because ESCRT is a conserved multifunctional complex, the presence of ESCRT genes in the genome does not necessarily suggest its involvement in cytokinesis. To determine whether ESCRT is primordial core machinery for eukaryotic cytokinesis, we explored ESCRT functions in the acidothermophilic unicellular red alga *Cyanidioschyzon merolae* that branched early in eukaryotic evolution (Yoon et al., 2004, 2006). In addition to the phylogenetical position, *C. merolae* provides an excellent experimental system. The cell (∼2 μm in diameter) has a simple structure (Kuroiwa, 1998). The genome (16.5 Mb, 5335 genes) has been completely sequenced (Matsuzaki et al., 2004; Nozaki et al., 2007). Genetic transformation is feasible (Ohnuma et al., 2008; Fujiwara et al., 2013), and a light and dark cycle highly synchronizes cell cycle progression and thus the timing of cytokinesis in a population (Suzuki et al., 1994; Supplementary Figure S1A).

Unlike other algae and plants, *C. merolae* does not have a rigid cell wall. It divides through membrane furrowing at the equator (constriction stage) that takes several minutes, followed by scission of the intercellular bridge (abscission stage), a stage that completes within a minute (Figure 1B; Supplementary Figures S1A,B). *C. merolae* lacks the actomyosin contractile ring and septins (Figure 1A). The actin gene does not seem to be expressed in *C. merolae*, and staining with phalloidin, which detects F-actin, is negative (Suzuki et al., 1995; Takahashi et al., 1995; Matsuzaki et al., 2004). Moreover, no myosin heavy chain gene or septin genes are present in the *C. merolae* genome (Matsuzaki et al., 2004). The only protein that has been linked to *C. merolae* cytokinesis is elongation factor (EF) 1α, which accumulates at the cleavage furrow (Figure 1B, Supplementary Figure S2; Imoto et al., 2011), as observed in *Tetrahymena* (Numata et al., 2000) and sea urchin eggs (Fujimoto and Mabuchi, 2010). Sea urchin EF1α bundles actin filaments and maintains the contractile ring structure (Fujimoto and Mabuchi, 2010). However, in *C. merolae*, actin filaments are probably absent and thus the function of EF1α in cytokinesis is unclear.

In this study, we investigated localization of ESCRT proteins in *C. merolae* by immunofluorescence and examined the effects of a dominant-negative mutant of VPS4 on cytokinesis. Five homologs of ESCRT-III proteins (CHMP1, CHMP2, and CHMP4–6), ALIX, and VPS4 localized at the intercellular bridge before cytokinetic abscission. ALIX also located close to the cleavage furrow early in the constriction stage. The expression of mutant VPS4 caused abscission failure, indicating that ESCRT mediates cytokinetic abscission in *C. merolae*.

## Results

**FIGURE 2:**
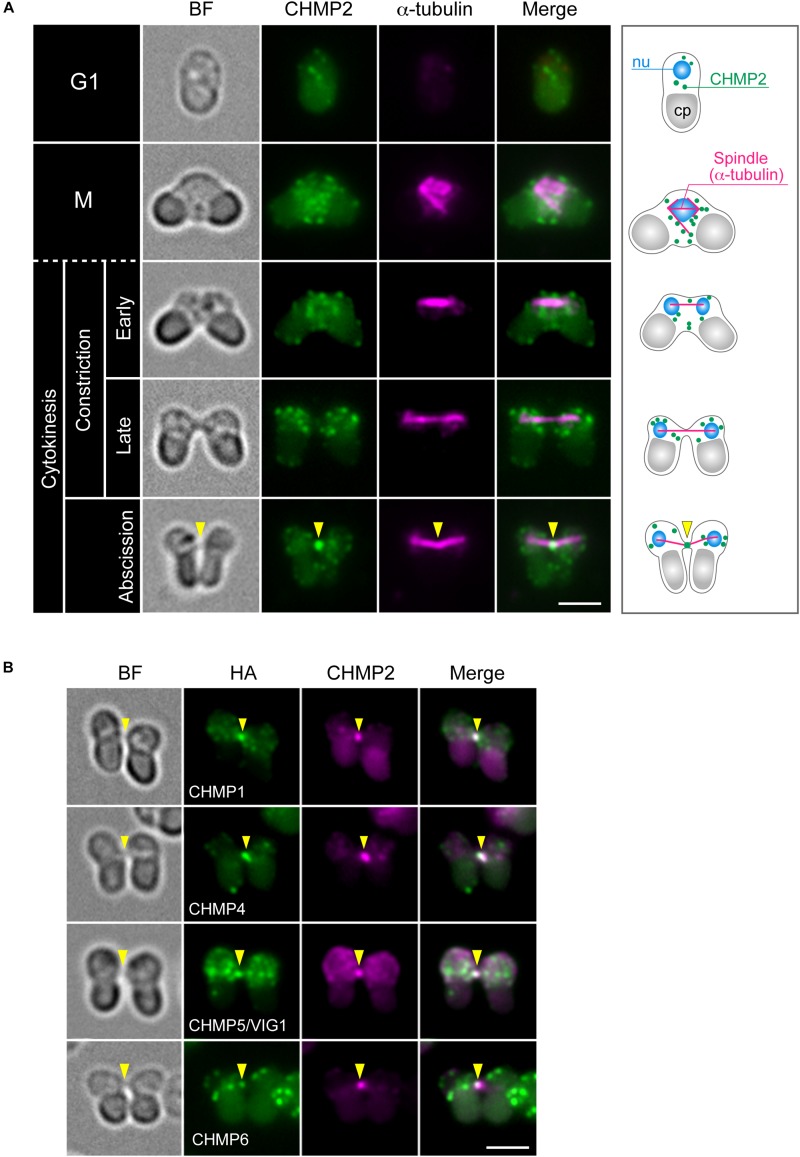
Localization of ESCRT-III proteins. **(A)** WT cells were fixed and labeled with anti-α-tubulin and anti-CHMP2 antibodies. A schematic representation is shown on the right. Because of the short duration of abscission, we found very few cells with the intercellular bridge. CHMP2 localization at the intercellular bridge was confirmed in 10 cells at the abscission stage (a total of three independent experiments). **(B)** Cells expressing CHMP1 (CMR340C)-HA, CHMP4-HA, CHMP5/VIG1-HA, or CHMP6-HA were fixed and labeled with anti-HA and anti-CHMP2 antibodies. Representative cells at the abscission stage are shown. *n* ≥ 5 cells were analyzed in two independent experiments for each strain. Arrowheads indicate the position of the intercellular bridge. BF, bright field. nu, cell nucleus; cp, chloroplast. Scale bars, 2 μm.

The *C. merolae* genome encodes homologs for 11 ESCRT proteins and ALIX (Table 1). We refer to these homologs according to the names of mammalian proteins except for the homolog of mammalian CHMP5, CHMP5/VIG1 (Vacuolar inheritance gene 1), which was previously characterized in *C. merolae* (Fujiwara et al., 2010; Yagisawa et al., 2018). To understand ESCRT functions in cytokinesis, we first examined the localization of ESCRT-III, the structure most directly involved in membrane deformation. We labeled CHMP2 using specific antibodies (Supplementary Figure S3A). In a synchronized culture under a light-dark cycle, the protein was expressed throughout the cell cycle with an increased level during the dark period (Supplementary Figure S3B). Immunofluorescence showed that CHMP2 localized on the punctate cytoplasmic structures and intercellular bridge of cytokinesis (Figure 2A). Next, we examined whether other ESCRT-III components localize with CHMP2 at the intercellular bridge using strains that ectopically expressed proteins fused to hemagglutinin (HA)-tags. *C. merolae* encodes two CHMP1 homologs (CMR340C and CMQ376C; Table 1). CHMP1-HA (CMR340C) localized at the intercellular bridge with CHMP2 (Figure 2B). CHMP1-HA (CMQ376C) was not expressed consistently with the lack of the expressed sequence tag (EST) of the native gene (data not shown; Matsuzaki et al., 2004). CHMP4-HA, CHMP5/VIG1-HA, and CHMP6-HA localized at the intercellular bridge with CHMP2 (Figure 2B).

**FIGURE 3:**
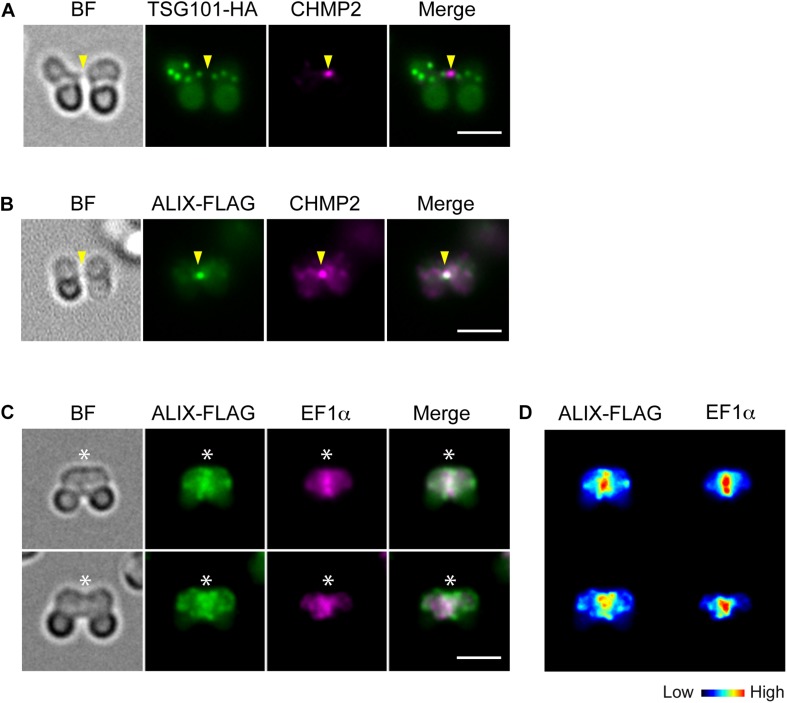
Localization of TSG101 and ALIX. **(A)** Images of a cell expressing TSG101-HA at the abscission stage. The fixed cell was labeled with anti-HA and anti-CHMP2 antibodies. Representative images are shown. Five cells at the abscission stage were analyzed in two independent experiments. **(B)** ALIX-FLAG cells were fixed and labeled with anti-FLAG and anti-CHMP2. Eight cells were analyzed in two independent experiments. **(C)** ALIX-FLAG cells were labeled with anti-FLAG and anti-EF1α antibodies, >30 cells were analyzed in each experiment (*n* = 2). **(D)** Heat map of the signal intensities in C. Arrowheads, the position of the intercellular bridge. Asterisks, the position of the cleavage furrow where ALIX-FLAG and EF1α localized. BF, bright field. Scale bars, 2 μm.

To further examine the involvement of ESCRT in *C. merolae* cytokinesis, we detected the localization TSG101, a major component of ESCRT-I, and ALIX. TSG101-HA was detected on the cytoplasmic puncta, but not on the intercellular bridge (Figure 3A). Although we also tested N-terminally tagged HA-TSG101, it was not expressed (data not shown). In contrast to TSG101, FLAG-tagged ALIX localized to the intercellular bridge (Figure 3B and Supplementary Figures S4A,B). During early constriction, ALIX-FLAG also located around the cleavage furrow (Figures 3C,D and Supplementary Figures S4A,B). The signals partially overlapped with those of EF1α (Figures 3C,D). In the other stages (G1, M, and late constriction), ALIX-FLAG was mainly localized close to the cell membrane and on some cytoplasmic structures (Supplementary Figures S4A,B).

Our attempts to knock out some ESCRT genes were unsuccessful, suggesting that ESCRT disruption is lethal in *C. merolae*. An ATPase-inactive dominant-negative mutant of VPS4 blocks cytokinetic abscission in mammalian cells (Carlton and Martin-Serrano, 2007; Morita et al., 2007). To further clarify the role of ESCRT in cytokinesis, we expressed the corresponding mutant VPS4 (E292Q) in *C. merolae* cells.

**FIGURE 4:**
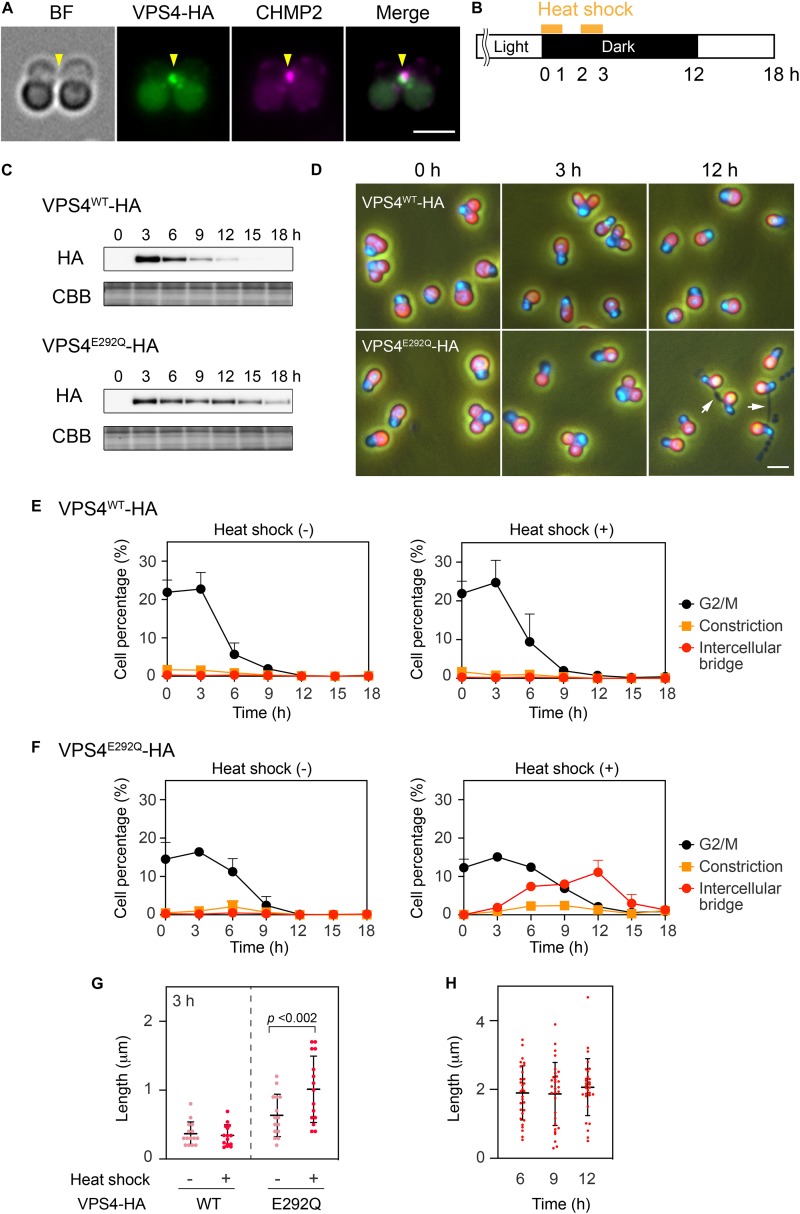
Heat shock induction of the mutant VPS4. **(A)** Immunofluorescence of cells expressing VPS4-HA under control of the native promoter sequence. The fixed cell was labeled with anti-HA and anti-CHMP2 antibodies. Representative images are shown. Five cells were analyzed in two independent experiments. Arrowheads, the position of the intercellular bridge. **(B)** Schematic representation of heat treatments performed in **(C–H)**. Cells harboring the *VPS4^WT^-HA* or *VPS4^E292Q^-HA* gene under the control of the heat-inducible promoter were synchronized by a 12 h light/12 h dark cycle at 42°C, the optimal growth temperature for wildtype cells. The culture was exposed to a higher temperature (50°C) for 1 h twice with a 1 h interval at the beginning of the dark period. **(C)** Immunoblotting of VPS4^WT^-HA and VPS4^E292Q^-HA. Proteins were extracted from cells collected at the indicated time point after the onset of the heat shocks. Total proteins were loaded in each lane and labeled with anti-HA antibodies. Some of the membrane was stained with Coomassie Brilliant Blue (CBB) as a loading control, *n* = 3. **(D)** DAPI staining of cells harboring heat-inducible *VPS4^WT^-HA* or *VPS4^E292Q^-HA*. The cells were fixed and stained before (0 h) and after the onset of heat shocks (3 and 12 h). Merged images of DAPI (blue), autofluorescence from chloroplasts (red), and phase contrast are shown. White arrows indicate cells with a long intercellular bridge. **(E) and (F)** Percentages of cells at the indicated cell cycle stages among cells harboring the *VPS4^WT^-HA* **(E)** or *VPS4^E292Q^-HA* **(F)**. “Intercellular bridge” includes cells at the abscission stage and those with an elongated intercellular bridge. **(G)** Length of the intercellular bridge at 3 h. Data from 15 cells (*n* = 5, three independent experiments) are shown in each column. Bars indicate the mean ± standard deviation. *p*, *p*-value of the Student’s *t*-test. **(H)** Length of the intercellular bridge in cells expressing VPS4^E292Q^-HA at the indicated time point. Thirty cells (*n* = 10, three independent experiments) were analyzed in each column. Scale bars, 2 μm.

**FIGURE 5:**
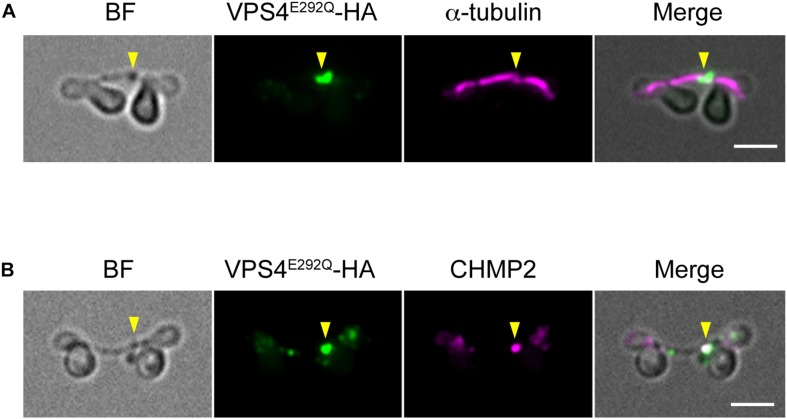
Localization of the spindle, mutant VPS4, and CHMP2. **(A,B)** Cells harboring heat-inducible *VPS4^E292Q^-HA* were fixed at 12 h after the start of heat shocks and then labeled with anti-HA and anti-α-tubulin **(A)** or anti-CHMP2 antibodies **(B)**. Representative images are shown. More than 30 cells with a long intercellular bridge (>1 μm) were imaged in each experiment (*n* = 3). The results showed that 94.5 ± 3.7% of the long intercellular bridges were positive for spindles, and 94.1 ± 5.3% were positive for VPS4^E292Q^-HA that colocalized with CHMP2. Arrowheads, locations of the VPS4E292Q-HA signal on the long intercellular bridge. BF, bright field. Scale bars, 2 μm.

When expressed under control of the native promoter sequence, VPS4-HA localized on the intercellular bridge (Figure 4A). To assess the effect of the mutation on cytokinesis, wild-type (^WT^) or the mutant (^E292Q^) *VPS4-HA* were expressed under the control of a heat-inducible promoter in the synchronized culture. The cells were subjected to heat treatments at the beginning of the dark period (G2/M phase, as shown in Figures 1B, 4B,C). VPS4^WT^-HA cells completed cell division in 12 h after the onset of heat shock, which was similar to untreated cells (Figures 4D,E). In contrast, induction of VPS4^E292Q^-HA accumulated cells with notably elongated intercellular bridges (Figures 4D,F–H). Most of these long intercellular bridges were spanned by the spindle (Figure 5A) and positive for VPS4^E292Q^-HA and CHMP2 (Figure 5B).

## Discussion

ESCRT potentially represents a component of the most ancient conserved machinery for cytokinetic abscission in eukaryotes. However, little is known about such ESCRT functions in eukaryotes other than in animals. In this study, we revealed that ESCRT is an essential component for cytokinetic abscission in *C. merolae*, an early diverged eukaryote.

**FIGURE 6:**
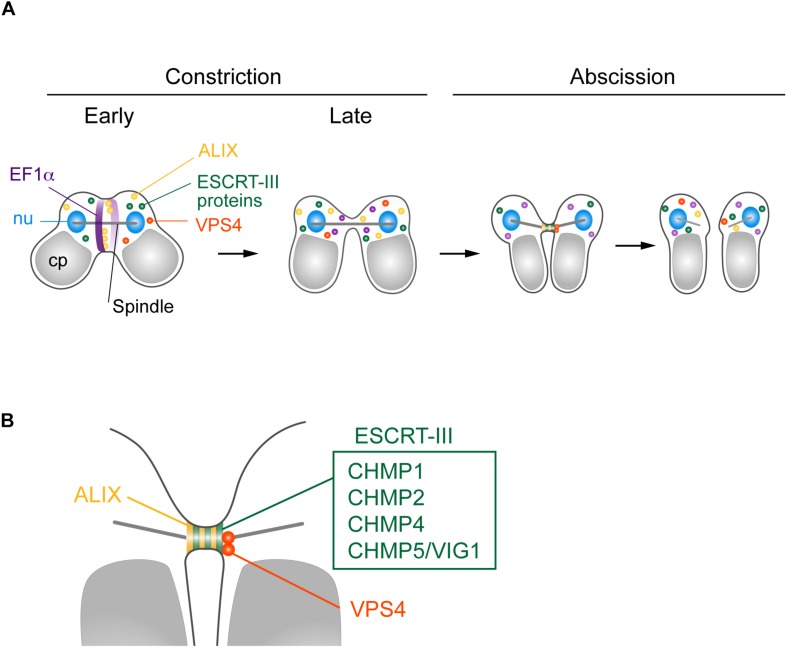
Suggested model for *C. merolae* cytokinesis. **(A)** EF1α and ALIX localized around the cleavage furrow during early constriction and are excluded during late constriction. ALIX, ESCRT-III proteins CHMP1, 2, 4, and 5, and VPS4 colocalized at the intercellular bridge in the abscission stage. **(B)** Enlarged image of the intercellular bridge before abscission.

We found that five ESCRT-III proteins, CHMP1 (CMR340C), CHMP2, CHMP4, CHMP5/VIG1, and CHMP6, localized at the intercellular bridge of *C. merolae* (Figures 6A,B). In mammalian cells, CHMP1–6, including its isoforms (Carlton and Martin-Serrano, 2007; Morita et al., 2007, 2010; Dukes et al., 2008; Yang et al., 2008; Bajorek et al., 2009; Elia et al., 2011; Guizetti et al., 2011; Carlton et al., 2012; Goliand et al., 2014; Christ et al., 2016), and IST1 (Agromayor et al., 2009; Goliand et al., 2018) localize at the midbody. *C. merolae* is devoid of genes encoding CHMP3 and IST1. In addition, ESCRT-III genes, except for *CHMP1*, exist as a single copy. Thus, ESCRT-III machinery in *C. merolae* is simpler in terms of protein composition. Electron microscopy has shown that mammalian ESCRT-III proteins either form or assist in forming a spiral of 17 nm-diameter filaments underlying the intercellular bridge membrane (Guizetti et al., 2011; Mierzwa et al., 2017; Schoneberg et al., 2017). The identification of such a structure is challenging in *C. merolae* because of the short duration of the abscission stage and small size of the intercellular bridge. Thus, further extensive studies are required to elucidate the structure involved in cytokinetic abscission.

*C. merolae* TSG101 appeared to be absent from the intercellular bridge. Mammalian ESCRT-I and ALIX localize at the midbody to separately target ESCRT-III (Christ et al., 2016). ESCRT-I depends on CHMP6 to recruit other ESCRT-III proteins, whereas the ALIX route does not (Christ et al., 2016). *C. merolae* CHMP6 resided at the intercellular bridge (Figures 6A,B). Thus, it potentially has a role unrelated to ESCRT-I. ESCRT-I is found in all major eukaryotic taxa but was secondarily lost in some species (Williams and Urbe, 2007; Leung et al., 2008). Although we cannot completely rule out the possibility that the addition of epitope-tags altered the localization of the protein or that the antibody could not react with the protein because of poor accessibility, the absence of TSG101 from the intercellular bridge may suggest a major role of ALIX in recruiting ESCRT-III. In mammalian cells, both ESCRT-I and ALIX are recruited by centrosome protein 55 kDa (CEP55), a midbody protein. However, CEP55 is absent in *C. elegans* and *Drosophila melanogaster*, although they depend on ESCRT for cytokinetic abscission (Green et al., 2013; Lie-Jensen et al., 2019). In *Drosophila*, ALIX is recruited to the midbody by Pavarotti, a homolog of human mitotic kinesin-like protein (MKLP) 1 (Lie-Jensen et al., 2019). In *C. merolae*, CEP55 or MKLP1 homologs have not been found. Thus, upstream mechanisms to recruit ESCRT appear to vary among organisms.

In contrast to TSG101, *C. merolae* ALIX was enriched at the intercellular bridge (Figures 6A,B). We also detected ALIX around the cleavage furrow early in the constriction stage (Figure 6A). Of related interest is CdvA, a scaffold protein for ESCRT-III in the archaea *Sulfolobus* (Lindas et al., 2008; Samson et al., 2011). It localizes to the mid-region of the cell, corresponding to membrane ingression sites from the beginning to final stages of cell division. The localization precedes that of ESCRT-III (Samson et al., 2011). Because *C. merolae* and eukaryotes other than Amorphea lack the actomyosin contractile ring, understanding the role of ALIX or ESCRT in the constriction stage would be of interest for future study.

We found that VPS4 localized to the intercellular bridge (Figures 6A,B). The phenotype of cells expressing VPS4^E292Q^-HA was strikingly similar to that observed after overexpression of VPS4, either WT or dominant-negative forms (Carlton and Martin-Serrano, 2007; Morita et al., 2007), or disruption of spastin (Connell et al., 2009) in mammalian cells. This phenotype is also reminiscent of that in the archaea *Sulfolobus* overexpressing truncated ESCRT-III proteins, which exhibit long intercellular bridges (Liu et al., 2017). Therefore, *C. merolae* VPS4 plays a pivotal role in scission of the intercellular bridge, as seen in these organisms. *C. merolae* VPS4^E292Q^-HA resided with CHMP2 on the long intercellular bridge, suggesting that the dynamics of ESCRT-III regulated by the AAA-ATPase activity of VPS4 are critical for cytokinetic abscission in this organism.

Finally, our data indicate that ESCRT mediates cytokinetic abscission in eukaryotic cells that lack the contractile ring and septins, and in the eukaryotic intercellular bridge that is considerably smaller than that of mammalian cells. In mammalian cells, the contractile ring is required for midbody formation (Hu et al., 2012). The polymerization state of actin controls midbody maturation which is essential for the appropriate assembly of ESCRT-III (Terry et al., 2018). The clearance of F-actin from the intercellular bridge after the furrow closure is also a limiting step in ESCRT-III recruitment (Fremont et al., 2016). Septins function in both the contractile ring and intercellular bridge. They are essential for maturation and stabilization of the intercellular bridge as well as proper ESCRT-III assembly (Renshaw et al., 2014; Addi et al., 2018; Karasmanis et al., 2019). The inhibitory effects of F-actin on ESCRT-III recruitment, and the role of septins in ESCRT-III assembly may be confined to animals or eukaryotic groups in which cell division is dependent on the contractile ring. The mammalian midbody is >1 μm in diameter (Mullins and Biesele, 1977; Green et al., 2012), and ESCRT-III is targeted for >40 min before abscission (Stoten and Carlton, 2018). However, in *C. merolae*, the intercellular bridge is ∼200 nm in diameter and requires less than 1 min to be cleaved (Supplementary Figure S1B). Thus, *C. merolae* appears to control cytokinetic abscission more simply than in mammalian cells. Importantly, regardless of these differences and the phylogenetic distance, ESCRT components mediate cytokinetic abscission in these organisms.

In summary, we demonstrate that five ESCRT-III proteins, ALIX, and VPS4 localize at abscission sites to mediate cytokinetic abscission in *C. merolae*. We also show that ESCRT functions in cytokinesis of an organism that lacks the contractile ring and septins. The fact that ESCRT mediates cytokinesis in archaea, animals, and the early diverged red alga *C. merolae* supports the idea that ESCRT is the primordial machinery for cytokinetic abscission in eukaryotes. We expect that exploring other lineages of eukaryotes that undergo ESCRT-mediated cytokinetic abscission and characterization of their mechanisms should further advance our understanding of the conserved mechanisms and evolution of eukaryotic cytokinesis.

## Materials and Methods

### Cell Culture

*C. merolae* wildtype (10D; Toda et al., 1998) and transformant cells were grown in MA2 medium (Ohnuma et al., 2008) at 30°C under continuous light (30 μE⋅m^–2^⋅s^–1^). To synchronize cell division, the cells (OD_750_ = 2–6) were diluted to OD_750_ = 0.4 in 2 × Allen’s medium (Allen, 1959) and subjected to a 12 h light (100 μE⋅m^–2^⋅s^–1^)/12 h dark cycle at 42°C with bubbling air (300 ml/min). Heat treatments were applied by shifting the synchronized culture to 50°C.

### Strain Generation

The primers and plasmids used to generate strains are listed in Supplementary Table S1. All strains except for the ALIX-FLAG strain were generated by integration of DNA fragments into the upstream region of the *URA5.3* gene (Fujiwara et al., 2015). To add tags to ESCRT proteins, plasmids containing transformation cassettes, which included the upstream (-2300 to -898 bp) of the *URA5.3* gene (CMK046C), genes encoding ESCRT proteins with their promoter region, 3 × HA-tag, the 3′ UTR of β-tubulin, and the *URA5.3* gene with the promoter region, were generated using an In-Fusion HD Cloning kit (Clontech). For *CHMP2*-, *CHMP4*-, and *CHMP5/VIG1-HA*, PCR products #1, #5 and one of #2–#4 (Supplementary Table S1) were used. For other ESCRT proteins, PCR products #6 and one of #7–#11 were used. Plasmids containing a heat shock promoter (Sumiya et al., 2014) and VPS4-HA were generated by fusing PCR products #12 and #13 using the In-Fusion HD cloning kit. The plasmid for dominant-negative *VPS4-HA* (E292Q; a mutation in conserved Walker B motif; Hanson and Whiteheart, 2005) was prepared by In-Fusion cloning of PCR product #14. Transformation of *C. merolae* was conducted as described previously (Ohnuma et al., 2008; Fujiwara et al., 2015). Briefly, the M4 strain (a point mutant of URA5.3; Minoda et al., 2004) was transformed with PCR-amplified cassettes from each plasmid (#15) using a polyethylene glycol-mediated method. The transformants were selected for uracil independence in starch placed on solidified MA2 medium (Fujiwara et al., 2013). Establishment of the strain ALIX-FLAG was performed following the procedures of Takemura et al., 2019a. PCR products #16 and #17, corresponding to the 3′-portion (from + 997 to + 2496, where +1 is the first base position of the initiation codon) and the 3′-downstream region (from +2497 to +3996) of the CMC051C (*ALIX*) ORF, respectively, were inserted into *Stu*I-digested pMKTf (Takemura et al., 2018) to construct the plasmid pMKTf-ALIX-Tagging. Subsequently, transformation cassette #18 was amplified from pMKTf-ALIX-Tagging by PCR and used to transform the uracil-auxotroph T1 strain (Taki et al., 2015) as described previously (Takemura et al., 2019a). Transformants were selected on uracil-free MA2 plates using the top starch method as described previously (Takemura et al., 2019b).

### Generation of an Antibody Against *C. merolae* CHMP2

DNA fragments encoding the CMB008C and pQE80 expression vector (Qiagen) were amplified by PCR with the primers listed in Supplementary Table S2. The fragments were fused and circularized using the In-Fusion HD cloning kit, resulting in a construct containing the six-histidine tag at the N-terminus of CMB008C. The recombinant proteins were purified with His-Trap columns (GE Healthcare Life Sciences) and used to raise antibodies in rats (T. K. craft, Ltd.).

### Time-Lapse Imaging

A synchronized culture at M phase was mounted on coverslips, which had pieces of surgical tape at the corners, and was then incubated for 30 min at room temperature. After removing excess medium, the coverslips were inverted and placed in glass-bottom dishes. The dishes were transferred into a chamber for live imaging (BZ-H3XD; Keyence) at 40°C. Images were obtained under a microscope (BZ-X700; Keyence) using a ×100 objective.

### Microscopy

For immunofluorescence, cells were fixed with methanol containing 1% formaldehyde and 10% DMSO at −20°C overnight. The fixed cells were centrifuged at 1500 × *g* at 4°C, washed once with cold methanol (−20°C), and then twice with PBS. For blocking, the cells were treated with either Blocking One (Nakarai Tesque) for 15 min at 4°C or 5% BSA for 30 min at 37°C. The antibody reaction was performed for 1 h at 4°C. Primary antibodies were diluted in PBS and used at the following dilutions: 1:500 for rat anti-CHMP2, 1:100 for rabbit anti-α-tubulin (Fujiwara et al., 2009), 1:500 for guinea pig anti-EF1α (Imoto et al., 2011), 1:1000 for mouse anti-HA (Clone 16B12; BioLegend), and 1:1000 for mouse anti-DYKDDDDK tag (to detect FLAG-tag; Clone 1E6; Wako). Fluorescent secondary antibodies (Thermo Fisher Scientific) were diluted in PBS and applied at 1:1000 for Alexa Fluor 488 and 1:100 for Alexa Fluor 555. DNA was stained with 1 μg/ml 4′,6-diamidino-2-phenylindole (DAPI). Images were acquired under the BZ-X700 fluorescence microscope using the × 100 objective. For Alexa Fluor 488, the GFP filter was used. The emission filter of the TRITC filter was changed to XF3022 (580DF30; Omega Optical) for Alexa Fluor 555 to avoid signals of chloroplast autofluorescence. To analyze the length of intercellular bridges, cells were fixed with 1% glutaraldehyde and imaged under the BZ-X700 microscope using the × 100 objective. The length was measured using ImageJ software (Schneider et al., 2012). For Figure 4D, cells were fixed with 1% glutaraldehyde and stained with 1 μg/ml DAPI. Images were obtained under a fluorescence microscope (BX51; Olympus) with a × 40 objective and CCD camera (C7780, Hamamatsu Photonics). The following filter sets were used: U-MWU2 (Olympus) for DAPI and U-MWIG2 (Olympus) for chloroplast autofluorescence. Heat maps of the signal intensities were generated in Image Lab software (Bio-Rad). All images were adjusted for contrast using Photoshop software (Adobe Systems).

### Immunoblotting

*C. merolae* cells were collected by centrifugation at 1500 × *g* at room temperature. The cell pellets were resuspended in 2 × SDS sample buffer (100 μm Tris, pH 6.8, 12% 2-mercaptoethanol, 4% SDS, and 20% glycerol) and incubated for 3 min at 95°C. After centrifugation at 15000 × *g* for 5 min at 4°C, the protein concentration in the supernatant was measured using an XL-Bradford kit (Aproscience). Total proteins (5 μg) were separated on polyacrylamide gels and then transferred to PVDF membranes. The membranes were blocked with 5% dry skim milk. The antibodies were diluted in 5% dry skim milk and used at the following dilutions: rat anti-CHMP2 (1:10000 for Supplementary Figure S3A and 1:2000 for Supplementary Figure S3B), rabbit anti-H3S10Ph (1:2000; Merk-Millipore), and mouse anti-HA (1:5000; Clone 16B12, BioLegend). Secondary antibodies were HRP-conjugated anti-rat, anti-rabbit, or anti-mouse IgG (1:20000; Thermo Fisher scientific). The signals were detected using ECL Prime (GE Healthcare) and the imaging system ImageQuant LAS-4000mini (for Supplementary Figure S3B; GE Healthcare) or ChemiDoc Touch (Bio-Rad).

## Data Availability Statement

The datasets generated for this study are available on reasonable request to the corresponding author.

## Author Contributions

FY and TF formulated the concept, designed the study, performed the experiments, analyzed and interpreted the data, and drafted the manuscript. TT, YK, and NS performed the experiments, analyzed and interpreted the data, and drafted the manuscript. NS performed the experiments and interpreted the data. SN, YI, OM, and KT designed the study and interpreted the data. SM, HK, and TK contributed to the concept, designed the study, interpreted the data, and drafted the manuscript.

## Conflict of Interest

The authors declare that the research was conducted in the absence of any commercial or financial relationships that could be construed as a potential conflict of interest.

## References

[B1] Addi C. Bai J. Echard A. (2018). Actin, microtubule, septin and ESCRT filament remodeling during late steps of cytokinesis. *Curr. Opin. Cell Biol.* 50 27–34. 10.1016/j.ceb.2018.01.007 29438904

[B2] Agromayor M. Carlton J. G. Phelan J. P. Matthews D. R. Carlin L. M. Ameer-Beg S. (2009). Essential role of hIST1 in cytokinesis. *Mol. Biol. Cell* 20 1374–1387. 10.1091/mbc.E08-05-0474 19129480 PMC2649264

[B3] Allen M. B. (1959). Studies with *Cyanidium caldarium*, an anomalously pigmented Chlorophyta. *Arch. Microbiol.* 32 270–277. 10.1007/bf00409348 13628094

[B4] Bajorek M. Morita E. Skalicky J. J. Morham S. G. Babst M. Sundquist W. I. (2009). Biochemical analyses of human IST1 and its function in cytokinesis. *Mol. Biol. Cell* 20 1360–1373. 10.1091/mbc.E08-05-0475 19129479 PMC2649257

[B5] Burki F. (2014). The eukaryotic tree of life from a global phylogenomic perspective. *Cold Spring Harb. Perspect. Biol.* 6:a016147. 10.1101/cshperspect.a016147 24789819 PMC3996474

[B6] Campsteijn C. Vietri M. Stenmark H. (2016). Novel ESCRT functions in cell biology, spiraling out of control? *Curr Opin Cell Biol.* 41 1–8. 10.1016/j.ceb.2016.03.008 27031044

[B7] Carlton J. G. Caballe A. Agromayor M. Kloc M. Martin-Serrano J. (2012). ESCRT-III governs the aurora B-mediated abscission checkpoint through CHMP4C. *Science* 336 220–225. 10.1126/science.1217180 22422861 PMC3998087

[B8] Carlton J. G. Martin-Serrano J. (2007). Parallels between cytokinesis and retroviral budding, a role for the ESCRT machinery. *Science* 316 1908–1912. 10.1126/science.1143422 17556548

[B9] Carvalho A. Desai A. Oegema K. (2009). Structural memory in the contractile ring makes the duration of cytokinesis independent of cell size. *Cell* 137 926–937. 10.1016/j.cell.2009.03.021 19490897

[B10] Christ L. Wenzel E. M. Liestol K. Raiborg C. Campsteijn C. Stenmark H. (2016). ALIX and ESCRT-I/II function as parallel ESCRT-III recruiters in cytokinetic abscission. *J. Cell Biol.* 212 499–513. 10.1083/jcb.201507009 26929449 PMC4772496

[B11] Connell J. W. Lindon C. Luzio J. P. Reid E. (2009). Spastin couples microtubule severing to membrane traffic in completion of cytokinesis and secretion. *Traffic* 10 42–56. 10.1111/j.1600-0854.2008.00847.x 19000169 PMC2709849

[B12] Cross F. R. Umen J. G. (2015). The *chlamydomonas* cell cycle. *Plant J.* 82 370–392. 10.1111/tpj.12795 25690512 PMC4409525

[B13] Dukes J. D. Richardson J. D. Simmons R. Whitley P. (2008). A dominant-negative ESCRT-III protein perturbs cytokinesis and trafficking to lysosomes. *Biochem. J.* 411 233–239. 10.1042/bj20071296 18076377

[B14] Elia N. Sougrat R. Spurlin T. A. Hurley J. H. Lippincott-Schwartz J. (2011). Dynamics of endosomal sorting complex required for transport (ESCRT) machinery during cytokinesis and its role in abscission. *Proc. Natl. Acad. Sci. U.S.A.* 108 4846–4851. 10.1073/pnas.1102714108 21383202 PMC3064317

[B15] Fremont S. Hammich H. Bai J. Wioland H. Klinkert K. Rocancourt M. (2016). Oxidation of F-actin controls the terminal steps of cytokinesis. *Nat. Commun.* 8:14528. 10.1038/ncomms14528 28230050 PMC5331220

[B16] Fujimoto H. Mabuchi I. (2010). Elongation factors are involved in cytokinesis of sea urchin eggs. *Genes Cells* 15 123–135. 10.1111/j.1365-2443.2009.01370.x 20059555

[B17] Fujiwara T. Kanesaki Y. Hirooka S. Era A. Sumiya N. Yoshikawa H. (2015). A nitrogen source-dependent inducible and repressible gene expression system in the red alga *Cyanidioschyzon merolae*. *Front. Plant Sci.* 6:657. 10.3389/fpls.2015.00657 26379685 PMC4549557

[B18] Fujiwara T. Kuroiwa H. Yagisawa F. Ohnuma M. Yoshida Y. Yoshida M. (2010). The coiled-coil protein VIG1 is essential for tethering vacuoles to mitochondria during vacuole inheritance of *Cyanidioschyzon merolae*. *Plant Cell* 22 772–781. 10.1105/tpc.109.070227 20348431 PMC2861457

[B19] Fujiwara T. Ohnuma M. Yoshida M. Kuroiwa T. Hirano T. (2013). Gene targeting in the red alga *Cyanidioschyzon merolae*, single- and multi-copy insertion using authentic and chimeric selection markers. *PLoS One* 8:e73608. 10.1371/journal.pone.0073608 24039997 PMC3764038

[B20] Fujiwara T. Yoshida Y. Kuroiwa T. (2009). Synchronization of cell nuclear, mitochondrial and chloroplast divisions in the unicellular red alga *Cyanidioschyzon merolae*. *Cytologia* 74:1.

[B21] Garcia-Salcedo J. A. Perez-Morga D. Gijon P. Dilbeck V. Pays E. Nolan D. P. (2004). A differential role for actin during the life cycle of *Trypanosoma brucei*. *EMBO J.* 23 780–789. 10.1038/sj.emboj.7600094 14963487 PMC381002

[B22] Goliand I. Adar-Levor S. Segal I. Nachmias D. Dadosh T. Kozlov M. M. (2018). Resolving ESCRT-III spirals at the intercellular bridge of dividing cells using 3D STORM. *Cell Rep.* 24 1756–1764. 10.1016/j.celrep.2018.07.051 30110633

[B23] Goliand I. Nachmias D. Gershony O. Elia N. (2014). Inhibition of ESCRT-II-CHMP6 interactions impedes cytokinetic abscission and leads to cell death. *Mol. Biol. Cell* 25 3740–3748. 10.1091/mbc.E14-08-1317 25232011 PMC4230781

[B24] Green R. A. Mayers J. R. Wang S. Lewellyn L. Desai A. Audhya A. (2013). The midbody ring scaffolds the abscission machinery in the absence of midbody microtubules. *J. Cell Biol.* 203 505–520. 10.1083/jcb.201306036 24217623 PMC3824018

[B25] Green R. A. Paluch E. Oegema K. (2012). Cytokinesis in animal cells. *Annu. Rev. Cell Dev. Biol.* 28 29–58. 10.1146/annurev-cellbio-101011-155718 22804577

[B26] Guizetti J. Schermelleh L. Mantler J. Maar S. Poser I. Leonhardt H. (2011). Cortical constriction during abscission involves helices of ESCRT-III-dependent filaments. *Science* 331 1616–1620. 10.1126/science.1201847 21310966

[B27] Hanson P. I. Whiteheart S. W. (2005). AAA+ proteins, have engine, will work. *Nat. Rev. Mol. Cell Biol.* 6 519–529. 10.1038/nrm1684 16072036

[B28] Henson J. H. Ditzler C. E. Germain A. Irwin P. M. Vogt E. T. Yang S. (2017). The ultrastructural organization of actin and myosin II filaments in the contractile ring, new support for an old model of cytokinesis. *Mol. Biol. Cell* 28 613–623. 10.1091/mbc.E16-06-0466 28057763 PMC5328620

[B29] Hosein R. E. Williams S. A. Haye K. Gavin R. H. (2003). Expression of GFP-actin leads to failure of nuclear elongation and cytokinesis in *Tetrahymena thermophila*. *J. Eukaryot. Microbiol.* 50 403–408. 10.1111/j.1550-7408.2003.tb00261.x 14733431

[B30] Hu C. K. Coughlin M. Mitchison T. J. (2012). Midbody assembly and its regulation during cytokinesis. *Mol. Biol. Cell* 23 1024–1034. 10.1091/mbc.E11-08-0721 22278743 PMC3302730

[B31] Imoto Y. Nishida K. Yagisawa F. Yoshida Y. Ohnuma M. Yoshida M. (2011). Involvement of elongation factor-1α in cytokinesis without actomyosin contractile ring in the primitive red alga *Cyanidioschyzon merolae*. *Cytologia* 76 431–437. 10.1508/cytologia.76.431

[B32] Iwaki T. Onishi M. Ikeuchi M. Kita A. Sugiura R. Giga-Hama Y. (2007). Essential roles of class E Vps proteins for sorting into multivesicular bodies in *Schizosaccharomyces pombe*. *Microbiology* 153 2753–2764. 10.1099/mic.0.2007/006072-0 17660439 PMC2885615

[B33] Karasmanis E. P. Hwang D. Nakos K. Bowen J. R. Angelis D. Spiliotis E. T. (2019). A septin double ring controls the spatiotemporal organization of the escrt machinery in cytokinetic abscission. *Curr. Biol.* 29 2174–2182. 10.1016/j.cub.2019.05.050 31204162 PMC6620605

[B34] Kuroiwa T. (1998). The primitive red algae *Cyanidium caldarium* and *Cyanidioschyzon merolae* as model system for investigating the dividing apparatus of mitochondria and plastids. *Bioessays* 20 344–354. 10.1002/(sici)1521-1878(199804)20:4<344::aid-bies11>3.0.co;2-2

[B35] Leung K. F. Dacks J. B. Field M. C. (2008). Evolution of the multivesicular body ESCRT machinery; retention across the eukaryotic lineage. *Traffic* 9 1698–1716. 10.1111/j.1600-0854.2008.00797.x 18637903

[B36] Lie-Jensen A. Ivanauskiene K. Malerod L. Jain A. Tan K. W. Laerdahl J. K. (2019). Centralspindlin recruits ALIX to the midbody during cytokinetic abscission in drosophila via a mechanism analogous to virus budding. *Curr. Biol.* 29 3538–3548. 10.1016/j.cub.2019.09.025 31607533

[B37] Lindas A. C. Karlsson E. A. Lindgren M. T. Ettema T. J. Bernander R. (2008). A unique cell division machinery in the Archaea. *Proc. Natl. Acad. Sci. U.S.A.* 105 18942–18946. 10.1073/pnas.0809467105 18987308 PMC2596248

[B38] Liu J. Gao R. Li C. Ni J. Yang Z. Zhang Q. (2017). Functional assignment of multiple ESCRT-III homologs in cell division and budding in *Sulfolobus islandicus*. *Mol. Microbiol.* 105 540–553. 10.1111/mmi.13716 28557139

[B39] Makarova K. S. Yutin N. Bell S. D. Koonin E. V. (2010). Evolution of diverse cell division and vesicle formation systems in Archaea. *Nat. Rev. Microbiol.* 8 731–741. 10.1038/nrmicro2406 20818414 PMC3293450

[B40] Matsuzaki M. Misumi O. Shin I. T. Maruyama S. Takahara M. Miyagishima S. Y. (2004). Genome sequence of the ultrasmall unicellular red alga *Cyanidioschyzon merolae* 10D. *Nature* 428 653–657. 10.1038/nature02398 15071595

[B41] Mierzwa B. E. Chiaruttini N. Redondo-Morata L. von Filseck J. M. Konig J. Larios J. (2017). Dynamic subunit turnover in ESCRT-III assemblies is regulated by Vps4 to mediate membrane remodelling during cytokinesis. *Nat. Cell Biol.* 19 787–798. 10.1038/ncb3559 28604678 PMC5493987

[B42] Minoda A. Sakagami R. Yagisawa F. Kuroiwa T. Tanaka K. (2004). Improvement of culture conditions and evidence for nuclear transformation by homologous recombination in a red alga, *Cyanidioschyzon merolae* 10D. *Plant Cell Physiol.* 45 667–671. 10.1093/pcp/pch087 15215501

[B43] Mishra M. Kashiwazaki J. Takagi T. Srinivasan R. Huang Y. Balasubramanian M. K. (2013). In vitro contraction of cytokinetic ring depends on myosin II but not on actin dynamics. *Nat. Cell Biol.* 15 853–859. 10.1038/ncb2781 23770677

[B44] Morita E. Colf L. A. Karren M. A. Sandrin V. Rodesch C. K. Sundquist W. I. (2010). Human ESCRT-III and VPS4 proteins are required for centrosome and spindle maintenance. *Proc. Natl. Acad. Sci. U.S.A.* 107 12889–12894. 10.1073/pnas.1005938107 20616062 PMC2919903

[B45] Morita E. Sandrin V. Chung H. Y. Morham S. G. Gygi S. P. Rodesch C. K. (2007). Human ESCRT and ALIX proteins interact with proteins of the midbody and function in cytokinesis. *EMBO J.* 26 4215–4227. 10.1038/sj.emboj.7601850 17853893 PMC2230844

[B46] Muller S. Jurgens G. (2016). Plant cytokinesis-No ring, no constriction but centrifugal construction of the partitioning membrane. *Semin. Cell Dev. Biol.* 53 10–18. 10.1016/j.semcdb.2015.10.037 26529278

[B47] Mullins J. M. Biesele J. J. (1977). Terminal phase of cytokinesis in D-98S cells. *J. Cell Biol.* 73 672–684. 10.1083/jcb.73.3.672 873994 PMC2111417

[B48] Nishihama R. Onishi M. Pringle J. R. (2011). New insights into the phylogenetic distribution and evolutionary origins of the septins. *Biol. Chem.* 392 681–687. 10.1515/BC.2011.086 21824002 PMC3951473

[B49] Nozaki H. Takano H. Misumi O. Terasawa K. Matsuzaki M. Maruyama S. (2007). A 100%-complete sequence reveals unusually simple genomic features in the hot-spring red alga *Cyanidioschyzon merolae*. *BMC Biol.* 5:28. 10.1186/1741-7007-5-28 17623057 PMC1955436

[B50] Numata O. Kurasawa Y. Gonda K. Watanabe Y. (2000). Tetrahymena elongation factor-1 alpha is localized with calmodulin in the division furrow. *J. Biochem.* 127 51–56. 10.1093/oxfordjournals.jbchem.a022583 10731666

[B51] Ohnuma M. Yokoyama T. Inouye T. Sekine Y. Tanaka K. (2008). Polyethylene glycol (PEG)-mediated transient gene expression in a red alga. *Cyanidioschyzon merolae* 10D. *Plant Cell Physiol.* 49 117–120. 10.1093/pcp/pcm157 18003671

[B52] Otegui M. S. Verbrugghe K. J. Skop A. R. (2005). Midbodies and phragmoplasts, analogous structures involved in cytokinesis. *Trends Cell Biol.* 15 404–413. 10.1016/j.tcb.2005.06.003 16009554 PMC3677513

[B53] Otto J. J. Schroeder T. E. (1990). Association of actin and myosin in the contractile ring. *Ann. N. Y. Acad. Sci.* 582 179–184. 10.1111/j.1749-6632.1990.tb21678.x 2192594

[B54] Pasha S. N. Meenakshi I. Sowdhamini R. (2016). Revisiting myosin families through large-scale sequence searches leads to the discovery of new myosins. *Evol. Bioinform. Online* 12 201–211. 10.4137/EBO.S39880 27597808 PMC5006635

[B55] Pollard T. D. (2017). Nine unanswered questions about cytokinesis. *J. Cell Biol.* 216 3007–3016. 10.1083/jcb.201612068 28807993 PMC5626534

[B56] Reichl E. M. Ren Y. Morphew M. K. Delannoy M. Effler J. C. Girard K. D. (2008). Interactions between myosin and actin crosslinkers control cytokinesis contractility dynamics and mechanics. *Curr. Biol.* 18 471–480. 10.1016/j.cub.2008.02.056 18372178 PMC2361134

[B57] Renshaw M. J. Liu J. Lavoie B. D. Wilde A. (2014). Anillin-dependent organization of septin filaments promotes intercellular bridge elongation and Chmp4B targeting to the abscission site. *Open Biol.* 4:130190. 10.1098/rsob.130190 24451548 PMC3909275

[B58] Samson R. Y. Obita T. Freund S. M. Williams R. L. Bell S. D. (2008). A role for the ESCRT system in cell division in archaea. *Science* 322 1710–1713. 10.1126/science.1165322 19008417 PMC4121953

[B59] Samson R. Y. Obita T. Hodgson B. Shaw M. K. Chong P. L. Williams R. L. (2011). Molecular and structural basis of ESCRT-III recruitment to membranes during archaeal cell division. *Mol. Cell.* 41 186–196. 10.1016/j.molcel.2010.12.018 21255729 PMC3763469

[B60] Schneider C. A. Rasband W. S. Eliceiri K. W. (2012). NIH Image to ImageJ, 25 years of image analysis. *Nat. Methods* 9 671–675. 10.1038/nmeth.2089 22930834 PMC5554542

[B61] Schoneberg J. Lee I. H. Iwasa J. H. Hurley J. H. (2017). Reverse-topology membrane scission by the ESCRT proteins. *Nat. Rev. Mol. Cell Biol.* 18 5–17. 10.1038/nrm.2016.121 27703243 PMC5198518

[B62] Schuh A. L. Audhya A. (2014). The ESCRT machinery, from the plasma membrane to endosomes and back again. *Crit. Rev. Biochem. Mol. Biol* 49 242–261. 10.3109/10409238.2014.881777 24456136 PMC4381963

[B63] Sebe-Pedros A. Grau-Bove X. Richards T. A. Ruiz-Trillo I. (2014). Evolution and classification of myosins, a paneukaryotic whole-genome approach. *Genome Biol. Evol.* 6 290–305. 10.1093/gbe/evu013 24443438 PMC3942036

[B64] Spitzer C. Schellmann S. Sabovljevic A. Shahriari M. Keshavaiah C. Bechtold N. (2006). The *Arabidopsis* elch mutant reveals functions of an ESCRT component in cytokinesis. *Development* 133 4679–4689. 10.1242/dev.02654 17090720

[B65] Stoten C. L. Carlton J. G. (2018). ESCRT-dependent control of membrane remodelling during cell division. *Semin. Cell Dev. Biol.* 74 50–65. 10.1016/j.semcdb.2017.08.035 28843980 PMC6015221

[B66] Sumiya N. Fujiwara T. Kobayashi Y. Misumi O. Miyagishima S. Y. (2014). Development of a heat-shock inducible gene expression system in the red alga *Cyanidioschyzon merolae*. *PLoS One* 9:e111261. 10.1371/journal.pone.0111261 25337786 PMC4206486

[B67] Suzuki K. Ehara T. Osafune T. Kuroiwa H. Kawano S. Kuroiwa T. (1994). Behavior of mitochondria, chloroplasts and their nuclei during the mitotic cycle in the ultramicroalga *Cyanidioschyzon merolae*. *Eur. J. Cell Biol.* 63 280–288. 8082652

[B68] Suzuki K. Kawazu T. Mita T. Takahashi H. Itoh R. Toda K. (1995). Cytokinesis by a contractile ring in the primitive red alga *Cyanidium caldarium* RK-1. *Eur. J. Cell Biol.* 67 170–178. 7664758

[B69] Takahashi H. Takano H. Yokoyama A. Hara Y. Kawano S. Toh-e A. (1995). Isolation, characterization and chromosomal mapping of an actin gene from the primitive red alga *Cyanidioschyzon merolae*. *Curr. Genet.* 28 484–490. 10.1007/bf00310820 8575024

[B70] Takemura T. Imamura S. Kobayashi Y. Tanaka K. (2018). Construction of a selectable marker recycling system and the use in epitope tagging of multiple nuclear genes in the unicellular red alga *Cyanidioschyzon merolae*. *Plant Cell Physiol.* 59 2308–2316. 10.1093/pcp/pcy156 30099537

[B71] Takemura T. Imamura S. Kobayashi Y. Tanaka K. (2019a). Multiple modification of chromosomal loci using URA5.3 selection marker in the unicellular red alga *Cyanidioschyzon merolae*. *Bioprotocol* 9:7 10.21769/BioProtoc.3204 PMC7854268 33655000

[B72] Takemura T. Kobayashi Y. Imamura S. Tanaka K. (2019b). Top starch plating method for the efficient cultivation of unicellular red alga *Cyanidioschyzon merolae*. *Bioprotocol* 9:4 10.21769/BioProtoc.3172 PMC7854263 33654978

[B73] Taki K. Sone T. Kobayashi Y. Watanabe S. Imamura S. Tanaka K. (2015). Construction of a URA5.3 deletion strain of the unicellular red alga *Cyanidioschyzon merolae*, A backgroundless host strain for transformation experiments. *J. Gen. Appl. Microbiol.* 61 211–214. 10.2323/jgam.61.211 26582291

[B74] Terry S. J. Dona F. Osenberg P. Carlton J. G. Eggert U. S. (2018). Capping protein regulates actin dynamics during cytokinetic midbody maturation. *Proc. Natl. Acad. Sci. U.S.A.* 115 2138–2143. 10.1073/pnas.1722281115 29439200 PMC5834733

[B75] Toda K. Takano H. Miyagishima S. Kuroiwa H. Kuroiwa T. (1998). Characterization of a chloroplast isoform of serine acetyltransferase from the thermo-acidiphilic red alga *Cyanidioschyzon merolae*. *Biochim. Biophys. Acta* 1403 72–84. 10.1016/s0167-4889(98)00031-7 9622597

[B76] Williams R. L. Urbe S. (2007). The emerging shape of the ESCRT machinery. *Nat. Rev. Mol. Cell Biol.* 8 355–368. 10.1038/nrm2162 17450176

[B77] Wloga D. Strzyzewska-Jowko I. Gaertig J. Jerka-Dziadosz M. (2008). Septins stabilize mitochondria in *Tetrahymena thermophila*. *Eukaryot. Cell* 7 1373–1386. 10.1128/EC.00085-08 18586950 PMC2519767

[B78] Wu J. Q. Ye Y. Wang N. Pollard T. D. Pringle J. R. (2010). Cooperation between the septins and the actomyosin ring and role of a cell-integrity pathway during cell division in fission yeast. *Genetics* 186 897–915. 10.1534/genetics.110.119842 20739711 PMC2975288

[B79] Yagisawa F. Imoto Y. Fujiwara T. Miyagishima S. (2018). “Single-membrane-bound organelles: division and inheritance,” in *Cyanidioschyzon merolae*, eds Kuroiwa T. (Singapore: Springer), 235–249. 10.1007/978-981-10-6101-1_15

[B80] Yamamoto M. Nishikawa T. Kajitani H. Kawano S. (2007). Patterns of asexual reproduction in *Nannochloris bacillaris* and *Marvania geminata* (Chlorophyta, Trebouxiophyceae). *Planta* 226 917–927. 10.1007/s00425-007-0538-7 17520279

[B81] Yamazaki T. Owari S. Ota S. Sumiya N. Yamamoto M. Watanabe K. (2013). Localization and evolution of septins in algae. *Plant J.* 74 605–614. 10.1111/tpj.12147 23398289

[B82] Yang D. Rismanchi N. Renvoise B. Lippincott-Schwartz J. Blackstone C. Hurley J. H. (2008). Structural basis for midbody targeting of spastin by the ESCRT-III protein CHMP1B. *Nat. Struct. Mol. Biol.* 15 1278–1286. 10.1038/nsmb.1512 18997780 PMC2593743

[B83] Yoon H. S. Hackett J. D. Ciniglia C. Pinto G. Bhattacharya D. (2004). A molecular timeline for the origin of photosynthetic eukaryotes. *Mol. Biol. Evol.* 21 809–818. 10.1093/molbev/msh075 14963099

[B84] Yoon H. S. Muller K. M. Sheath R. G. Ott F. D. Bhattacharya D. (2006). Defining the major lineages of red algae (Rhodophyta). *J. Phycol.* 42 482–492. 10.1111/j.1529-8817.2006.00210.x

[B85] Zaremba-Niedzwiedzka K. Caceres E. F. Saw J. H. Backstrom D. Juzokaite L. Vancaester E. (2017). *Asgard archaea* illuminate the origin of eukaryotic cellular complexity. *Nature* 541 353–358. 10.1038/nature21031 28077874

[B86] Zhou Q. Hu H. Li Z. (2014). New insights into the molecular mechanisms of mitosis and cytokinesis in trypanosomes. *Int. Rev. Cell Mol. Biol.* 308 127–166. 10.1016/B978-0-12-800097-7.00004-X 24411171 PMC4374570

