# Supplementary material for: ESCRT Machinery Mediates Cytokinetic Abscission in the Unicellular Red Alga Cyanidioschyzon merolae
Source: Front Cell Dev Biol. 2020 Apr 3;8:169. doi: 10.3389/fcell.2020.00169 (PMC7169423; doi:10.3389/fcell.2020.00169)
Supplement: Supplementary file 1 [file Data_Sheet_1.pdf]

Supplementary Material

Table S1. Primers, templates, and plasmids used to generate *C. merolae* strains.

PCR product #	Description of the PCR product	Template	Primer	Primer sequence (5' to 3')	Plasmid cloned using the PCR [illegible]
1	Vector backbone	pU-APCCp vector (Fujiwara et al. 2015)	OFY387	AGTCATACAACAGTACTCAGATCGTTGAGGAACAATGAAAG	pTF216, 219, 220
			OFY388	TAAACTAGCTATTTATCTGGTACATATCATTCATAAGCACATGTTTTTG	
2	CMB008C (*CHMP2*) and the upstream region	*C. merolae* genome	OFY389	TACTGTTGTATGACTATACGCAGGGTTGATGGTTC	pTF219
			OFY390	GTTCGAATTTTGCAGCGGCGGTTTCCTTGGCGCCTGCACCGGATCCTTTTTTTAAATTCTCAAGGCGGAGC	
3	CMI044C (*CHMP4*) and the upstream region	*C. merolae* genome	OFY391	TACTGTTGTATGACTCAGCGGAGACCTTCGGCC	pTF220
			OFY392	GTTCGAATTTTGCAGCGGCGGTTTCCTTGGCGCCTGCACCGGATCCAATGCGTGGCTCCGCGATA	
4	CML153C (*CHMP5/VIG1*) and the upstream region	*C. merolae* genome	OFY393	TACTGTTGTATGACTCGACCGGTTCCCTATCGTC	pTF216
			OFY394	GTTCGAATTTTGCAGCGGCGGTTTCCTTGGCGCCTGCACCGGATCCGACCGCCATGCTGCGT	
5	HA-tag	pBSHAb-T3' (Ohnuma et al. 2008)	OFY395	CTGCAAAATTCGAACGCCAGCACATGGACAGCCGATCCCGGGCTATGTACCCATACGATGTTCCTGAC	pTF216, 219, 220
			OFY396	TAAATAGCTAGTTTAAGCGTAATCTGGAACGTCATAAGG	

6	Vector backbone with HA tag	pTF219	OFY471<br><br>OFY472	AGTCATACAACAGTACTCAGATCGTT<br><br>GGATCCGGTGCAGGCGC	pFY56-59, 62
7	CMO281C (*VPS4*) and the upstream region	*C. merolae* genome	OFY473<br><br>OFY474	TACTGTTGTATGACTTGGTTTCCATGCACTGTTGT<br><br>GCCTGCACCGGATCCAACGCACTCTTGGCCGAAA	pFY56
8	CMK136C (*TSG101*) and the upstream region	*C. merolae* genome	OFY475<br><br>OFY476	TACTGTTGTATGACTTCGCCGAATAGTCTTATTTCTGG<br><br>GCCTGCACCGGATCCCGTGTTGCCATCCAGCGTT	pFY57
9	CMQ376C (*CHMP1*) and the upstream region	*C. merolae* genome	OFY477<br><br>OFY478	TACTGTTGTATGACTTAATCGCCCATATCCCCTTG<br><br>GCCTGCACCGGATCCCGGCAACTGCAGTGCCCG	pFY58
10	CMR340C (*CHMP1*) and the upstream region	*C. merolae* genome	OFY479<br><br>OFY480	TACTGTTGTATGACTAGCGTTCTCCTTGCCTTGTC<br><br>GCCTGCACCGGATCCGGGTGCCGGCGCAGATAG	pFY59
11	CMQ184C (*CHMP6*) and the upstream region	*C. merolae* genome	OFY525<br><br>OFY526	TACTGTTGTATGACTTCGCAGTAGGCTCGTCGTAA<br><br>GCCTGCACCGGATCCGGCTGCTTCCGCAATGTG	pFY62
12	Upstream region of CMJ101C (Heat-inducible promoter)	*C. merolae* genome	OFY506<br><br>OFY507	TACTGTTGTATGACTCTTATAGCTTACGTGGCGGA<br><br>GAATCCCTGGTTCTCTCACA	pFY63, 68
13	Linearized vector with CMO281C (*VPS4*)-*HA*	pFY56	OFY508 (used with	GAGAACCAGGGATTCATGACTTCGTTTGTGGACAA	pFY63
14		pFY63	OFY509	TATTGATCAAGTGGATGCGC	pFY68

	Linearized vector containing the heat shock		OFY510	TCCACTTGATCAATAAAGATAATAGAG	
15	Transformation cassettes	pTF216, 219, 220 pFY56-59, 62, 63, 68	OFY397	CTTCAAGAAAAGAGGATCTTTTGCCGTGATGCC	-
			OFY398	CCCTAGCAGCTGACTGTATCTCTATTCTTAGGAAT	
16	The 3’-portion of CMC051C (*ALIX*)	*C. merolae* genome	TTC051F1	GACACGTGAATTTAAATAGGATCGCGGAAAGGCTACTCAAG	pMKTf-ALIX-Tagging
			TTC051R1	TGAAAATAAAGATTTTCAGGTGACGACCCTAGCGAAACTTC	
17	The 3’-downstream region of CMC051C (*ALIX*)	*C. merolae* genome	TTC051F2	AGGGATTGTGGCGCGACAGGTGAGGCCGTCGTTGTCGCT	pMKTf-ALIX-Tagging
			TTC051R2	GACACGTGAATTTAAATAGGTCTTGTCTGGACTTTCCTCGAA	
18	Transformation cassette	pMKTf-ALIX-Tagging	TTC051F1	GACACGTGAATTTAAATAGGATCGCGGAAAGGCTACTCAAG	-
			TTC051R2	GACACGTGAATTTAAATAGGTCTTGTCTGGACTTTCCTCGAA	

Table S2. Primers used to generate an expression vector for 6×His-CHMP2.

PCR Product	Template	Primer	Sequence (5' to 3')
Vector backbone	pQE-80L (Qiagen)	OFY535	GTGATGGTGATGGTGATGC
		OFY536	TAATTAGCTGAGCTTGGACTCC
CMB008C (*CHMP2*)	*C. merolae* genome	OFY537	CACCATCACCATCACATGGAAGCGCTGAAGAAAAT
		OFY538	AAGCTCAGCTAATTATTTTTTTAAATTCTCAAGGCGG

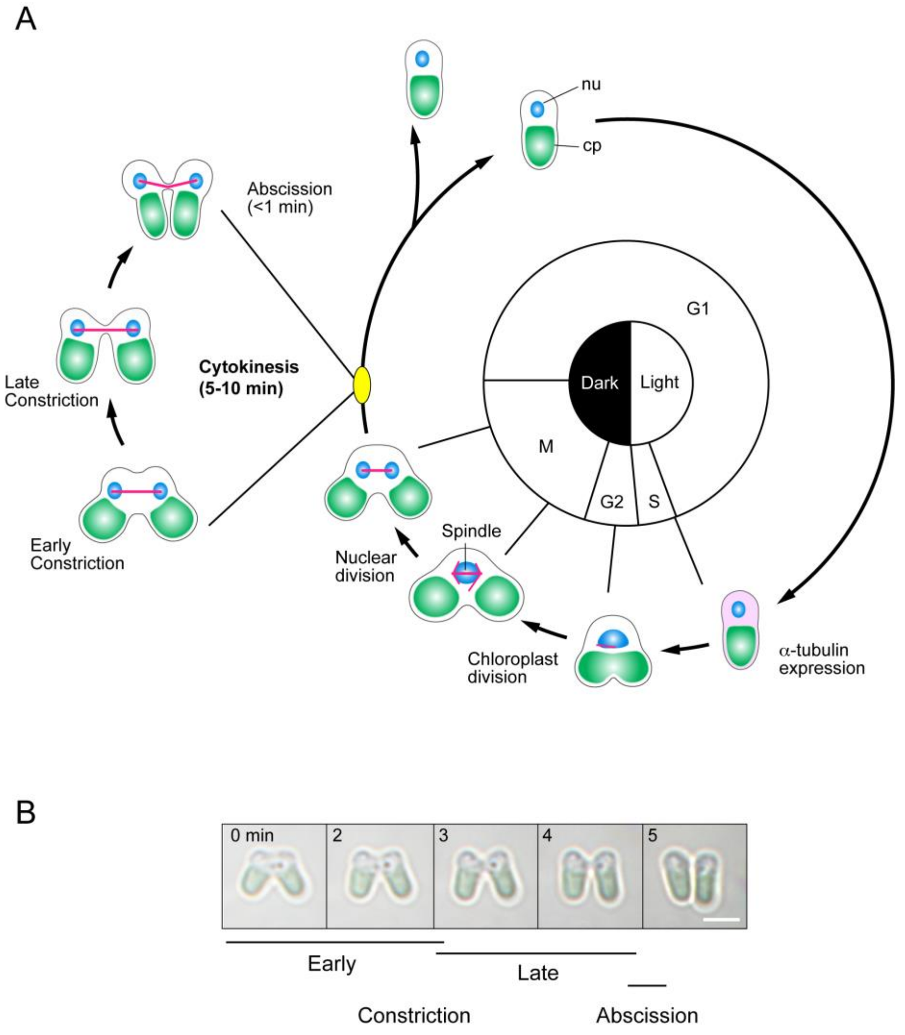

Figure S1. *C. merolae* cell cycle (A) A 12 h light/12 h dark cycle synchronized the cell division. α-tubulin is expressed in S phase to form the microtubules and the spindle. Organelle division proceeds cytokinesis. The cell divides once during the dark period. (B) Time-lapse images of a cell at cytokinesis. Scale bar, 2 μm.

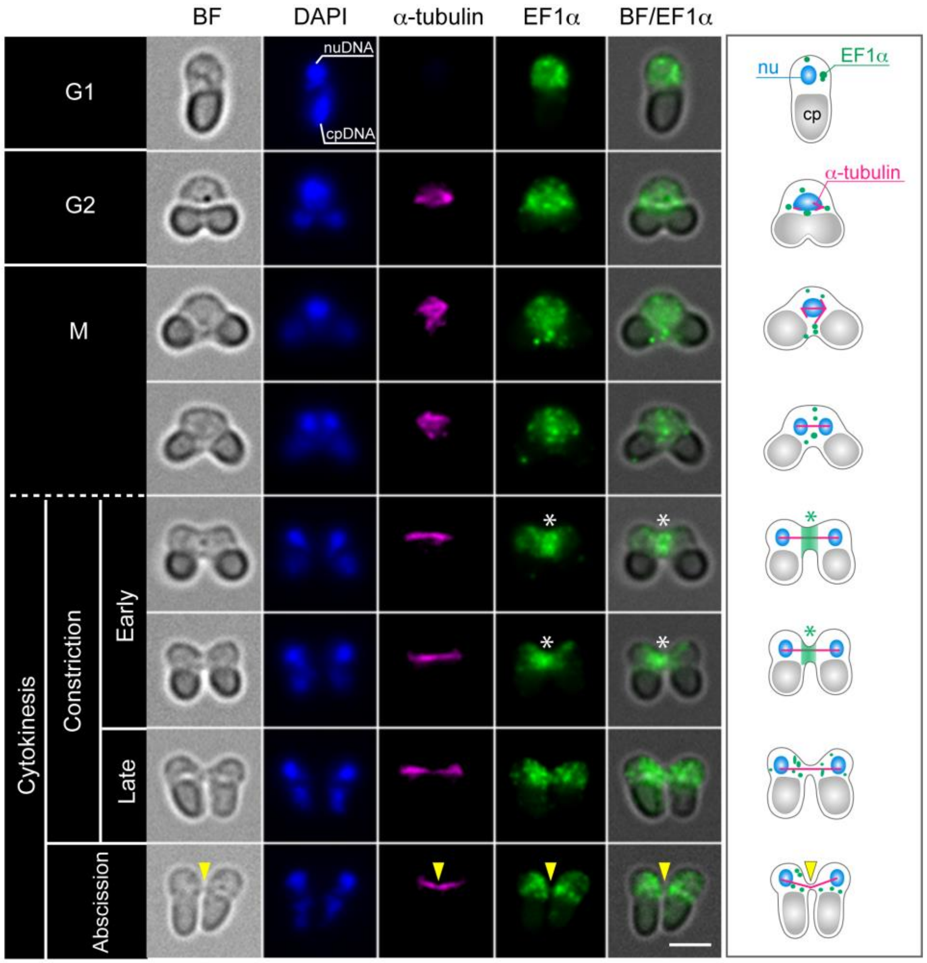

Figure S2. Dynamics of α-tubulin and EF1α during the cell cycle. Synchronized cells were labeled with DAPI, and anti-α-tubulin and anti-EF1α antibodies. α-tubulin was unexpressed in G1 phase. Microtubule formation and chloroplasts division started in G2 phase. In M phase, the spindle was formed in the cell nucleus to segregate nuclear DNA. After nuclear separation, cytokinesis began through membrane furrowing at the equator (constriction stage). During early constriction, EF1α formed a band at the cleavage furrow. Late in the constriction stage, the EF1α band was disassembled. Cytokinetic abscission occurred at a narrow intercellular bridge. The spindle was severed shortly before or concomitantly with membrane abscission. The exact timing for spindle severing was not elucidated, considering that the center of the spindle in mammalian cells is inaccessible to anti-α-tubulin antibodies (Hu et al. 2011). Asterisks indicate EF1α localized around the cleavage furrow. Arrowheads indicate positions of the intercellular bridge. BF, bright field. nu, cell nucleus; cp, chloroplast. Scale bar, 2 µm.

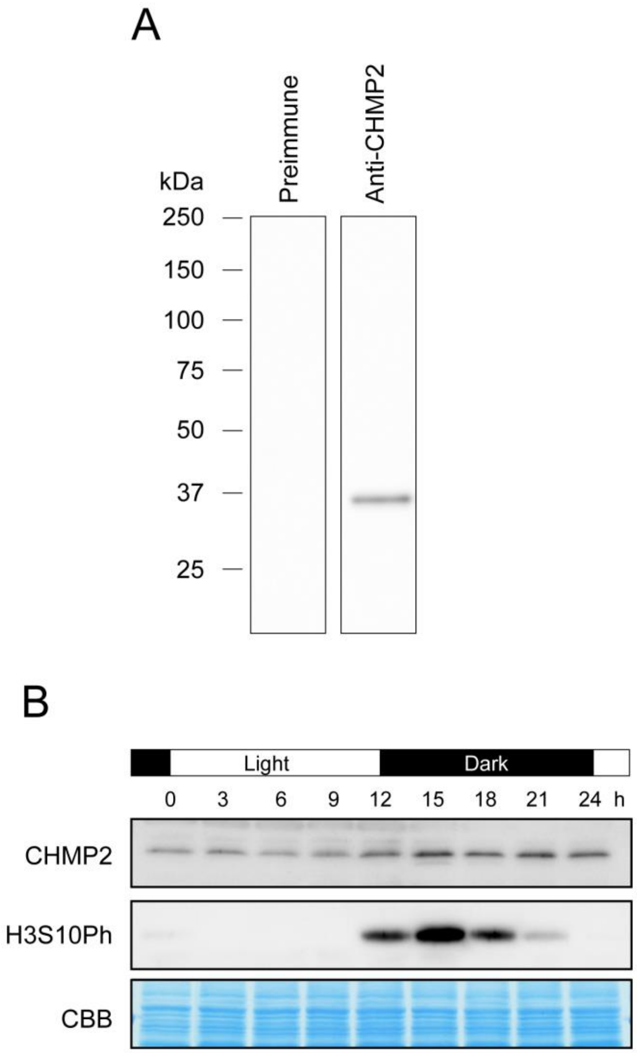

Figure S3. Immunoblotting with anti-CHMP2 antibodies. (A) Immunoblot using antibodies raised against *C. merolae* CHMP2. *C. merolae* total proteins were loaded in each lane. A 36.5 kDa band was detected (theoretical mass: 35.1 kDa). (B) Immunoblotting using anti-CHMP2 and anti-H3S10Ph antibodies (M phase marker; Fujiwara et al. 2013). Total proteins extracted from synchronized cells were loaded in each lane. Some of the membrane was stained with Coomassie Brilliant Blue (CBB) as a loading control. n=3.

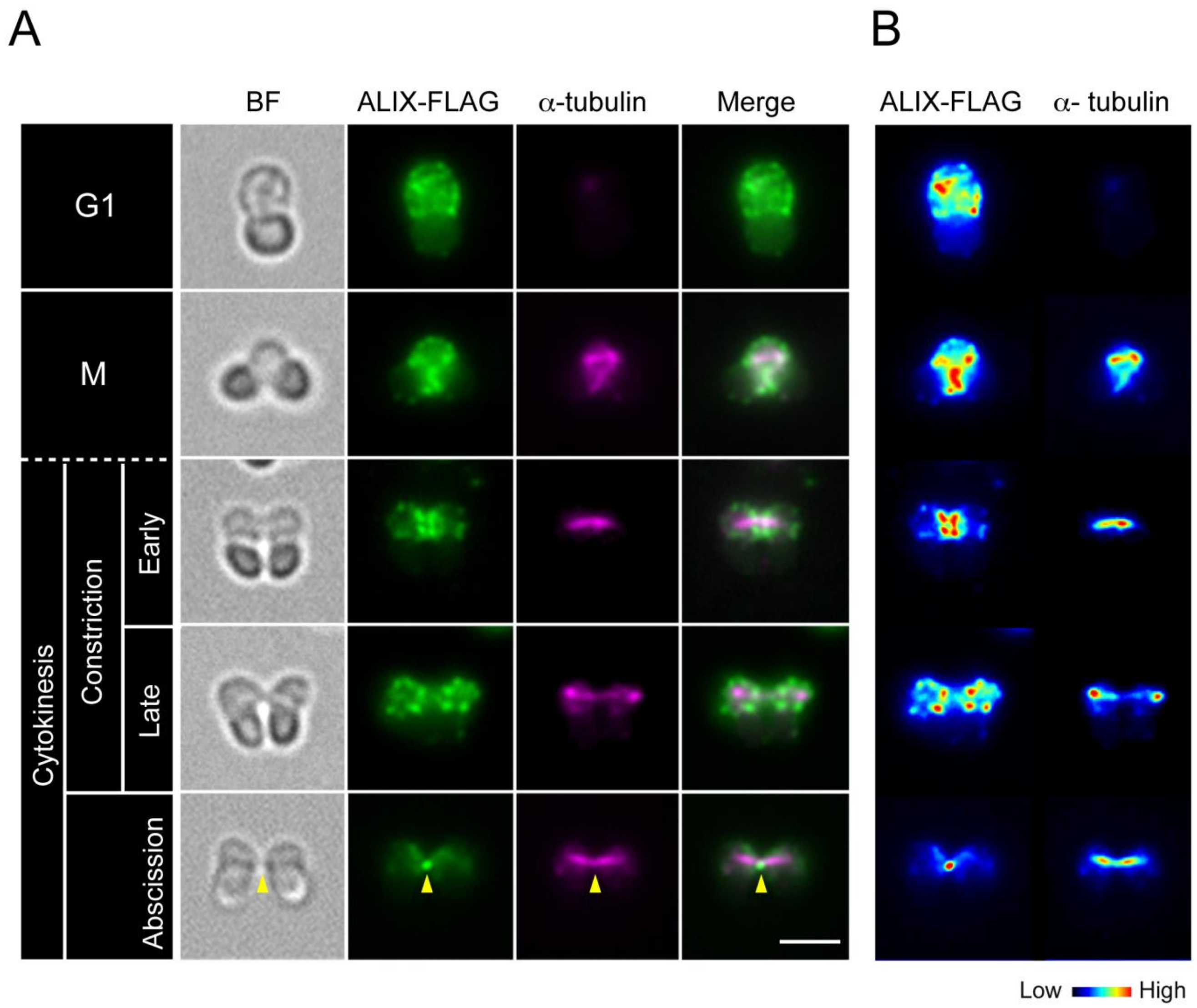

Figure S4. Localization of ALIX. (A) ALIX-FLAG cells were fixed and labeled with anti-FLAG and anti-α-tubulin antibodies. (B) Heat map of the signal intensities in B. Arrowheads, the position of the intercellular bridge. BF, bright field. Scale bars, 2 μm.
